# Differential response to Janus kinase inhibitors in refractory livedoid vasculopathy: Biomarker-guided therapy may improve clinical response

**DOI:** 10.1016/j.jdin.2025.11.024

**Published:** 2025-12-10

**Authors:** Guoqun Yu, Jinjin Zhu, Yuan Zhang, Fan Yang, Jing Yang, Juan Tao

**Affiliations:** a Department of Dermatology, Union Hospital, Tongji Medical College, Huazhong University of Science and Technology (HUST), Wuhan, China; b Hubei Engineering Research Center for Skin Repair and Theranostics, Wuhan, China

**Keywords:** JAK activation, Janus kinase inhibitors, livedoid vasculopathy, off-label treatment, personalized therapy, refractory skin disease

*To the Editor:* The treatment of livedoid vasculopathy (LV) has been a significant clinical challenge, particularly in refractory cases, where conventional therapies such as anticoagulants, anabolic steroids, or thrombolytics, either alone or in combination, often yield suboptimal efficacy or cause undesirable side effects.^1^ Recent studies have highlighted the potential of Janus kinase (JAK) inhibitors as a novel therapeutic option for LV.^2^, ^3^, ^4^ Inspired by these, we prescribed off-label baricitinib (JAK1/2 inhibitor) to 3 patients with refractory LV and followed them for at least 9 months. The outcomes, however, varied in terms of skin lesion resolution and associated symptom improvement.

The first 2 patients demonstrated improvement within 3 to 4 weeks, whereas the third patient exhibited slower lesion resolution, with noticeable improvement occurring only after 10 weeks (Table I; Supplementary Fig 1, available via Mendeley at https://data.mendeley.com/datasets/m5wk5hhfyz/1). This variability is consistent with previous findings, where rapid responders typically show improvement within 3 weeks, while slower responders may require several months.^2^^,^^3^ Additionally, the first 2 patients achieved sustained remission during the 20-month and 12-month follow-up, respectively, whereas the third patient experienced recurrent left ankle pain within 3 months after baricitinib tapering.

**Table I:** Characteristics of patients treated with Janus kinase inhibitors for livedoid vasculopathy

Patient	Age (y)/sex	Body weight (kg)	Location of skin lesions	Duration of disease (mo)	Previous therapies	JAKi dosage	Remission time (wk)	Follow-up time (mo)
1	10/M	44	Ankle, dorsum of the foot	24	C, RL, TA	Baricitinib 2 mg bid	4	20
2	16/M	53	Lower leg, ankle, dorsum of the foot	12	C, R	Baricitinib 4 mg qd	3	12
3	54/M	75	Lower leg, ankle, dorsum of the foot	6	D, TC	Baricitinib 4 mg qd	10	5
						Tofacitinib 5 mg bid	6	4

*bid*, Twice a day; *C*, corticosteroid; *D*, dipyridamole; *JAKi*, Janus kinase inhibitors; *M*, male; *qd*, once a day; *R*, rivaroxaban; *RL*, red light; *TA*, topical antibiotics; *TC*, topical corticosteroid.

This heterogeneity in treatment response prompted us to hypothesize that it might be underpinned by differences in JAK pathway activation within the skin lesions. To investigate this potential correlation, we performed immunohistochemical staining to assess the activation status of phosphorylated JAK (pJAK) 1, JAK2 (pJAK2), and JAK3 (pJAK3) in paraffin-embedded tissue samples. We enrolled 11 with LV (including patient 2 and 3), 10 with atopic dermatitis (SCORAD [Scoring Atopic Dermatitis] ≥25, without any systemic treatment) as positive controls, and 8 healthy controls. The average optical density was calculated for each sample using Image J software. The results showed significantly elevated pJAK1, pJAK2, and pJAK3 levels in LV samples compared to healthy controls (*P* < .0001, *P* < .05, and *P* < .0001, respectively; Fig 1). Comparison with atopic dermatitis lesions revealed that LV shared comparable levels of pJAK1 and pJAK2, but exhibited a markedly higher level of pJAK3 (Fig 1).

**Fig 1:**
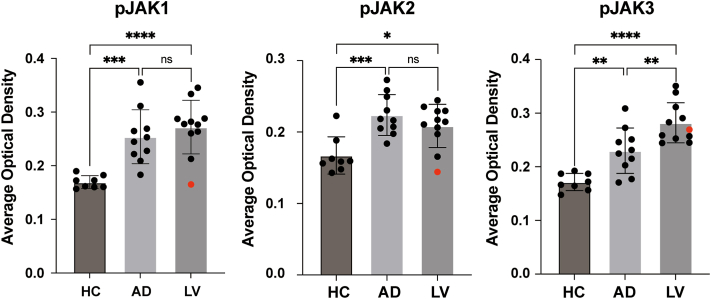
The comparison of pJAK1, pJAK2, and pJAK3 levels in LV, AD, and HCs. Average optical density (AOD) values were subjected to one-way analysis of variance and Tukey's test for multiple comparisons (∗*P* < .05, ∗∗*P* < .01, ∗∗∗*P* < .001, ∗∗∗∗*P* < .0001). The *red dot* represents the result of patient 3.

Notably, patient 3 exhibited lower pJAK1 and pJAK2 levels but markedly elevated pJAK3 expression (Fig 1; Supplementary Fig 2, available via Mendeley at https://data.mendeley.com/datasets/m5wk5hhfyz/1), which might explain his suboptimal response to baricitinib. These findings may account for the patient’s inadequate clinical improvement. Accordingly, we transitioned the patient to tofacitinib (5 mg bid), which targets JAK3 in addition to JAK1. This adjustment led to substantial lesion resolution and pain relief by week 6 (Supplementary Fig 1, available via Mendeley at https://data.mendeley.com/datasets/m5wk5hhfyz/1).

Although previous studies have suggested the therapeutic potential of JAK inhibitors in LV, their use remains a tentative approach due to the lack of robust supporting evidence. Here, we detected 11 skin lesions of LV and found that most of them had significantly elevated JAK activation, suggesting that Janus kinase inhibitors may represent a potentially effective option for refractory LV. Additionally, our observations raise the hypothesis that variability in JAK pathway activation could contribute to differential treatment responses. We propose that assessing JAK activation profiles might help refine treatment strategies, but further studies with larger cohorts are needed to validate this approach, particularly in exploratory or off-label settings.

## Conflicts of interest

None disclosed.
